# Cryoballoon Ablation for Treatment of Atrial Fibrillation in a Chinese Population: Five-Year Outcomes and Predictors of Recurrence After a Single Procedure

**DOI:** 10.3389/fcvm.2022.836392

**Published:** 2022-04-27

**Authors:** Xiongbiao Chen, Yu Xia, Yuan Lin, Xiaofeng Li, Chun Wang, Yanjun Chen, Pihua Fang, Jun Liu

**Affiliations:** ^1^Department of Cardiology, Peking University Shenzhen Hospital, Shenzhen, China; ^2^State Key Laboratory of Cardiovascular Disease, Cardiac Arrhythmia Center, Fuwai Hospital, National Center for Cardiovascular Diseases, Chinese Academy of Medical Sciences and Peking Union Medical College, Beijing, China; ^3^Department of General Practice, School of General Practice and Continuing Education, Capital Medical University, Beijing, China

**Keywords:** atrial fibrillation, cryoballoon, pulmonary vein isolation, long-term, follow-up

## Abstract

**Background:**

The 5-year outcomes and predictors of atrial fibrillation (AF) recurrence following cryoballoon (CB) ablation in Chinese population remain scarce. Our aim was to report 5-year outcomes and predictors of AF recurrence following a single CB ablation procedure in a Chinese population.

**Methods:**

From December 2013 to August 2016, we included 256 consecutive patients (mean age: 58 ± 10.9 years old; female: 41.0%) with paroxysmal or persistent AF successfully underwent first-generation CB ablation at Fuwai hospital in this prospective study. All patients were followed at least 5 years or when there was recurrent AF. Independent predictors of AF recurrence were determined by Cox proportional hazards regression analysis.

**Results:**

The 5-year success rate after pulmonary vein isolation (PVI) by a single procedure was 59.4%. The recurrence rate was the highest (14.5%) within the first year after the index procedure, and then stabilized. Patients with paroxysmal AF had a higher incidence of freedom from AF recurrence than patients with persistent AF (63.2% vs. 36.4%, log-rank *P* < 0.01). The overall incidence of complications related to CB ablation was 7.8%. Phrenic nerve injury (PNI) was the most common complication, with an incidence of 3.5%, and patients with PNI were recovered within the 1-year follow-up. Only persistent AF (HR 1.72, 95%CI 1.028–2.854, *P* < 0.05) was significantly and independently associated with an increased risk of AF recurrence after adjusting for other factors.

**Conclusion:**

Pulmonary vein isolation using CB ablation was safe and effective with an acceptable complication and 5-year success rate in a Chinese population with AF, and persistent AF was the independent predictor for 5-year AF recurrence after a single CB ablation procedure.

## Introduction

Pulmonary vein (PV) isolation (PVI) is the cornerstone for the ablation of atrial fibrillation (AF) (1, 2). In terms of both efficacy and safety, PVI *via* cryoballoon (CB) ablation has been proven to be non-inferior to radiofrequency catheter ablation in treating patients with symptomatic drug-refractory AF (3). Furthermore, it also offers many potential advantages over radiofrequency catheter ablation, such as short procedure time and learning curve (4). Since CB was introduced into mainland China in 2013, the number of CB ablation in the treatment of AF has increased continually. A few studies have reported the long-term outcomes after PVI with CB for Chinese population with AF (5–7). In this study, our aim was to report the 5-year outcomes and predictors of recurrence in a Chinese population who have undergone the treatment of paroxysmal or persistent AF by CB ablation.

## Materials and Methods

### Study Population

For the population enrollment of this prospective study, consecutive patients with documented symptomatic and drug-refractory (failure of ≥ 1 class I or III antiarrhythmic drugs) paroxysmal or persistent AF who underwent PVI using first-generation CB at Fuwai Hospital from December 2013 to August 2016 were included. Patients with episodes of AF terminated spontaneously or cardioverted within 7 days were defined as paroxysmal AF, and those whose episodes lasting longer than 7 days and terminated within 1 year were defined as persistent AF (2). Exclusion criteria: (1) patients with heart valve disease; (2) patients with any contraindications for the procedure, including the presence of acute thrombosis or severe bleeding occurred within 8 weeks before procedure; (3) patients with previous ablation of AF; (4) patients with left atrium diameter (LAD) ≥ 50 mm; and (5) patients younger than 18 years old. All enrolled patients gave written consent for data collection and publication. The study was carried out in compliance with the Helsinki Declaration and approved by the local institutional ethics committee.

### Preprocedural Management

All patients gave written informed consent for the ablation procedure. The preprocedural management was performed as our previous report (8). Briefly, routine transthoracic echocardiography examination was used to evaluate the cardiac structure and function before the procedure, including LAD and left ventricular ejection fraction. Anatomic structure of PV and left atrium were also assessed by multi-detector computed tomographic scan and transesophageal echocardiography. All patients were anticoagulated with vitamin K antagonists for at least 4 weeks before the procedure, with international normalized ratio maintained between 2.0 and 3.0. Vitamin K antagonist was discontinued 3 days prior to the procedure, and bridged with low-molecular-weight heparin injected subcutaneously till the early morning of the procedure day. Antiarrhythmic drugs were also discontinued 3 days prior to the procedure excepting beta blockers.

### Cryoballoon Ablation Procedure

The CB ablation procedure was performed as our previous report (9). Briefly, the procedure was performed under local anesthesia and deep sedation using midazolam and fentanyl. A decapolar diagnostic catheter (C. R. Bard, Inc., NY, United States or St. Jude Medical Inc., MN, United States) and a bipolar catheter (St. Jude Medical Inc., MN, United States) were placed into coronary sinus and right ventricular apex, respectively. After a single transseptal puncture by BRK-1 needle and standard 8.5-Fr SL1 long sheath (Synaptic Medical Inc., Beijing, China), heparin (100 IU/kg) was injected through peripheral vein, and then activated clotting time was measured every 30 min to maintain it for 250–350 s by supplementing heparin. The anatomy and caliber of PVs were confirmed by performing selective PV angiography. The appropriate CB (Arctic Front, Medtronic Inc., MN, United States) and circular mapping catheter (Achieve, Medtronic, CA, United States) size were selected accordingly. If the maximum diameter of three or all PVs were < 22 mm, 23-mm CB and 15-mm circular mapping catheter were selected. Otherwise, 28-mm CB and 20-mm circular mapping catheter are preferred. After that, the transseptal sheath was exchanged with a 15-Fr steerable sheath (FlexCath, Medtronic Inc., MN, United States) by a stiff guidewire. The CB with circular mapping catheter was advanced into each PV ostium, and PV potentials were recorded by circular mapping catheter. After achieving occlusion of PV ostium indicated by the contrast retention in PV, each freezing cycle was usually applied for 240 s in each vein. Additional bonus application of freezing was delivered to achieve PVI if necessary. When the temperature of CB was declined to below −60°C, the freezing time should be terminated before 240 s to guarantee the safety of operation. Furthermore, according to the practical experience of operators, the freezing time might be prolonged or shortened when applicating bonus freezing cycle to achieve PVI. To prevent phrenic nerve injury (PNI) during ablation of right-sided PVs, the bipolar catheter was positioned into superior vena cava for pacing phrenic nerve (10 mA, 2 ms, 50/min) constantly. Once diaphragmatic motion was weakened, ablation was immediately ceased (10). The procedural endpoint was bidirectional electric isolation of all PVs. If procedural endpoint could not be achieved with CB alone, touch up ablations with conventional radiofrequency catheter were performed to achieve bilateral isolation of all PVs.

### Definition of Complications

According to current guideline, procedure complications were defined as those occurring during the procedure, within hospitalization period or 30-day post ablation, including pericardial tamponade/effusion, transient ischemic attack, ischemic or hemorrhagic stroke, PV stenosis etc (11).

### Postablation Management and Follow-Up

The postablation management and follow-up were performed as our previous report (8). Briefly, transthoracic echocardiography was performed to rule out pericardial effusion within 3 h after the procedure for all patients. Oral anticoagulation agent was re-initiated 48–72 h after the procedure and continued for 3 months, was determined by individual CHA_2_DS_2_-VASc scores (11). An antiarrhythmic drug was prescribed for all patients throughout the 3-month blanking period, and thereafter terminated if the patient was free of AF recurrence. The type of antiarrhythmic drug was resumed as that used before ablation procedure. Paroxysmal AF patients were given propafenone, and persistent AF patients or paroxysmal AF patients with coronary heart disease or chronic congestive heart disease were given amiodarone. Patients were scheduled for clinic follow-up at 3-months intervals within first year after the procedure and at 6-month intervals afterward. Electrocardiogram and 24-h Holter monitor were performed in each follow-up to detect atrial arrhythmia. In addition, patients were asked to immediately complete an additional outpatient visit when they had symptoms indicated AF recurrence. The AF recurrence was defined as any episode of documented atrial tachycardia, atrial flutter, and/or AF lasting at least 30 s after 90-dayblanking period.

### Statistical Analysis

Continuous variables were reported as mean ± standard deviation or median [quartile] according to their nominal distribution. Categorical variables were reported as counts (percentage). Recurrence-free survival was estimated by the Kaplan-Meier method, and the statistical difference between groups was compared by log-rank test. Cox proportional hazards model was used to estimate the hazard ratios (HRs) and corresponding 95% confidence intervals (CIs) for independent predictors of AF recurrence. All statistical analyses were performed by using R software, version 3.6.0 (R Foundation for Statistical Computing, Vienna, Austria). A 2-tailed *P*-value < 0.05 was considered as statistically significant.

## Results

### Baseline Clinical Characteristics

In total, 256 consecutive patients with mean age of 58 ± 10.9 years old underwent CB ablation for AF (paroxysmal AF of 87.1%, persistent AF of 12.9%), and 41.0% were female. The baseline clinical characteristics of all patients are shown in Table 1.

**TABLE 1:** Baseline characteristics.

	Overall (*n* = 256)
Age (years old)	58 ± 10.9
Female	105 (41.0%)
BMI (Kg/m^2^)	25 ± 3.2
**Types of AF**	
Paroxysmal AF	223 (87.1%)
Persistent AF	33 (12.9%)
History of AF (years)	36.0 [12.0, 60.0]
Hypertension	118 (46.1%)
Diabetes mellitus	32 (12.5%)
Previous TIA/stroke	27 (10.5%)
CHD	40 (15.6%)
CHF	10 (3.9%)
PVD	77 (30.1%)
LAD (mm)	37 ± 6.8
LVEDD (mm)	48 ± 5.3
LVEF (%)	65 ± 6.5
CHA_2_DS_2_-VASc score	1.9 ± 1.5
HAS-BLED score	1.2 ± 1.0

*Data are expressed as mean ± SD, n (%) or median [Q1, Q3].*

*AF, atrial fibrillation; BMI, body mass index; CHD, coronary heart diseases; CHF, chronic congestive heart failure; LAD, left atrial diameter; LVEDD, left ventricular end-diastolic diameter; LVEF, left ventricular ejection fraction; PVD, peripheral vascular diseases; TIA, transient ischemic attack.*

### Procedure Characteristics

The procedure characteristics are summary in Table 2. The mean procedure and fluoroscopy time were 95 ± 25.4 min and 32 ± 11.8 min, respectively. There were 1,045 PVs in 256 patients, of which 9 patients had a left common PV and 30 patients had a right middle PV. Additional touch up ablations with radiofrequency catheter were performed in 21 patients (8.2%) to achieve PVI for the reason that it is difficult to completely occlude and isolate PVs with critical angulation. 152 patients (59.4%) were treated with the 23-mm CB, and 97 patients (37.9%) with the 28-mm CB. A combination of both CBs was used in 7 (2.7%) patients. The mean nadir temperature of superior PV was 6°C lower than that of the same side inferior PV.

**TABLE 2:** Procedural characteristics.

	Overall (*n* = 256)
Number of all PVs	1,045
Procedure time (min)	95 ± 25.4
Fluoroscopy time (min)	32 ± 11.8
**Cryoballoon type**	
23-mm	152 (59.4%)
28-mm	97 (37.9%)
23-mm + 28-mm	7 (2.7%)
**Bi-direction isolation**	
LSPV	237 (96.0%)
LIPV	239 (96.8%)
LCPV (*n* = 9)	8 (89.9%)
RIPV	241 (94.1%)
RMPV (*n* = 30)	27 (90.0%)
RSPV	248 (96.9%)
**Freezing cycles**	
LSPV	2.5 ± 0.6
LIPV	2.4 ± 0.8
LCPV (*n* = 9)	3.0 ± 1.2
RIPV	2.4 ± 0.9
RMPV (*n* = 30)	1.3 ± 0.6
RSPV	2.3 ± 0.7
**Nadir temperature (°C)**	
LSPV	−54 ± 6.2
LIPV	−48 ± 5.5
LCPV (*n* = 9)	−53 ± 3.2
RIPV	−50 ± 8.3
RMPV (*n* = 30)	−41 ± 5.8
RSPV	−56 ± 5.5
**Mean freezing time (s)**	
LSPV	221 ± 37.6
LIPV	244 ± 26.5
LCPV (*n* = 9)	219 ± 25.3
RIPV	234 ± 36.5
RMPV (*n* = 30)	240 ± 31.5
RSPV	209 ± 46.0

*Data are expressed as mean ± SD or n (%).*

*PV, pulmonary vein; RIPV, right inferior pulmonary vein; LSPV, left superior pulmonary vein; LIPV, left inferior pulmonary vein; RSPV, right superior pulmonary vein; LCPV, left common pulmonary vein; RMPV, right middle pulmonary vein.*

### Complications

The overall incidence of procedural complications related to CB ablation was 7.8% in our study. PNI was the most common complication, and occurred in 9 patients (3.5%) with 23-mm CB, of which 8 patients had recovered within the procedure and 1 patient had recovered at the time of 9-month follow-up. Five patient (2.0%) developed mild pericardial effusion with no necessity of pericardial drainage, and had subsided before discharge. Six patients (2.3%) developed vascular complications, including 4 patients with pseudoaneurysm and 2 patients with femoral arteriovenous fistula, who recovered by treating conservatively. There was no fatal complication like stroke, atrio-esophageal fistula or death occurred.

### Clinical Follow-Up

**FIGURE 1:**
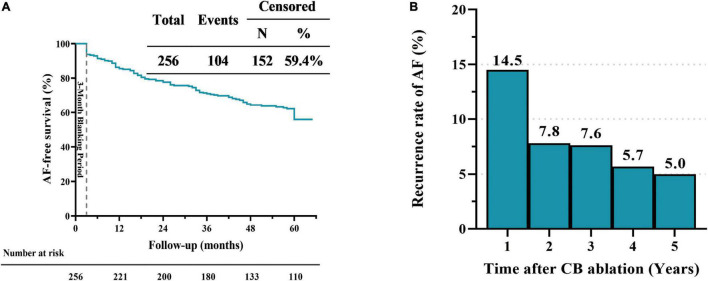
Five-year outcomes in patients with atrial fibrillation (AF). Kaplan–Meier curves showing AF-free survival curve after single ablation procedure performed using cryoballoon **(A)**. The histogram shows the initial recurrences rate of atrial fibrillation at one-year interval during the five-year follow-up after a single cryoballoon ablation procedure **(B)**.

**FIGURE 2:**
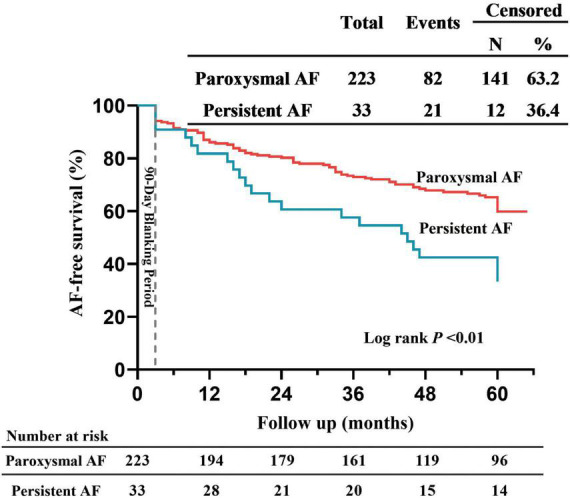
Comparing 5-year outcome in patients with paroxysmal and persistent AF. Kaplan–Meier curves for AF-free survival after single ablation procedure performed using cryoballoon.

The 5-year success rate after PVI by a single CB ablation procedure was 59.4% (Figure 1), with recurrences owing to atrial tachycardia (*n* = 14, 13.5%), atrial flutter (*n* = 8, 7.7%) and AF (*n* = 82, 78.8%). AF-free survival rates after PVI by a single CB ablation procedure were 85.5, 77.7, 70.1, and 64.4% at 1, 2, 3, and 4 years, respectively (Figure 1A). The AF recurrence rate was the highest (14.5%) in the first year after the index procedure, and declined to 7.8% in the 2 year, then stabilized (7.6, 5.7, and 5.0% in the third, fourth and fifth year, respectively; Figure 1B). AF-free survival rates after PVI by a single CB ablation procedure for patients with persistent AF were 81.8, 60.6, 57.6, and 42.4% at 1, 2, 3 and 4 years, respectively (Figure 2). The incidence of freedom from AF recurrence was significantly higher in patients with paroxysmal AF compared with those with persistent AF (63.2% vs. 36.4%, log-rank *P* < 0.01; Figure 2).

Out of 104 patients with AF recurrence, 18 patients underwent repeat ablation which was guided by a 3-dimensional electroanatomic mapping system after a median time of 36.5 [8.0, 47.5] months from the initial procedure. In these 18 patients, only 3 had no PV reconnection, who experienced recurrence owing to AT originating from superior vena cava. After a median time of 7.5 [3.0, 21.8] months from the initial procedure, 15 patients who had PV reconnection experienced AF recurrence. Among these patients, 38 (63.3%) out of 60 PVs showed reconnection (about 2.5 PVs reconnection per patient). In 38 reconnecting PVs, 14 (36.8%) were left superior PV, following by right superior PV (*n* = 11, 28.9%), right inferior PV (*n* = 9, 23.7%) and left inferior PV (*n* = 4, 10.5%).

### Predictors of Atrial Fibrillation Recurrence

As showed in Table 3, univariate Cox regression analysis indicated that persistent AF (HR 2.03, 95%CI 1.268–3.252, *P* < 0.01), LAD (HR 1.04, 95%CI 1.015–1.067, *P* < 0.01) and left common PV (HR 1.88, 95%CI 1.023–6.090, *P* < 0.05) were significantly associated with an increased risk of AF recurrence. To further confirm the independent prediction of these variables, only variables with *P*-value < 0.1 in univariate Cox regression analysis were included for stepwise multivariate analysis. Multivariate Cox regression analysis showed that only persistent AF (HR 1.72, 95%CI 1.028–2.854, *P* < 0.05) was significantly and independently associated with an increased risk of AF recurrence after adjusting for age, LAD, left ventricle end-diastolic diameter and left common PV (Table 3).

**TABLE 3:** Univariate and multivariate Cox regression analysis for predictors of AF recurrence.

	Uni-COX analysis			Multi-COX analysis		
	HR	95%CI	*P*-value	HR	95%CI	*P*-value
Age	1.06	0.989–1.024	0.093	1.02	0.992–1.046	0.180
Sex (Female)	1.03	0.693–1.518	0.899			
BMI	1.01	0.949–1.066	0.851			
Persistent AF	2.03	1.268–3.252	0.003	1.72	1.028–2.854	0.032
History of AF (years)	1.00	0.995–1.003	0.666			
Hypertension	0.87	0.593–1.287	0.494			
Diabetes mellitus	0.89	0.497–1.589	0.690			
Previous TIA/stroke	1.34	0.773–2.307	0.301			
CHD	1.48	0.914–2.384	0.111			
PVD	0.94	0.619–1.433	0.779			
CHF	1.31	0.534–3.224	0.554			
LAD	1.04	1.015–1.067	0.002	1.03	0.999–1.060	0.053
LVEDD	0.97	0.939–1.009	0.096	0.96	0.899–1.018	0.165
LVEF	1.02	0.984–1.047	0.356			
LCPV	1.88	1.023–6.090	0.045	1.40	0.784–7.322	0.125
RMPV	0.65	0.203–2.115	0.479			
CHA_2_DS_2_-VASc score	1.03	0.906–1.165	0.679			
HAS-BLED score	0.97	0.807–1.176	0.778			

*PV, pulmonary vein; RIPV, right inferior pulmonary vein; LSPV, left superior pulmonary vein; LIPV, left inferior pulmonary vein; RSPV, right superior pulmonary vein; LCPV, left common pulmonary vein; RMPV, right middle pulmonary vein.*

## Discussion

### Main Findings

This study reported the 5-year follow-up outcomes after single ablation procedure with first-generation CB in a Chinese population with AF. The major results of our study were as follows: (1) after a single CB ablation procedure, 59.4% of the patients were free of AF recurrence during 5-year follow-up (63.2% in paroxysmal and 36.4% in persistent AF); (2) the AF recurrence mainly occurred within the first year after the index procedure, and then it stabilized; (3) the incidence of procedure complications was 7.8%, and PNI was the most common complication with a rate of 3.5%; (4) persistent AF (HR 1.72, 95%CI 1.028–2.854, *P* < 0.05) was the significant and independent predictor of AF recurrence during 5-year follow-up.

### Five-Year Outcomes After Cryoballoon Ablation

So far, although several studies reported the five-year outcomes in patients treated with CB for AF, data on that in Chinese population remain scarce. Neumann et al. (12) reported the 5-year success rate was 53% in 163 patients from Europe with paroxysmal AF after PVI using first-generation CB. Studies also reported that the 5-year outcomes for PVI using the second-generation CB in patients with AF, and the success rate was 57.2–59.0% (13, 14). In our study, the 5-year success rate was 59.4% after a single CB ablation procedure in patients with paroxysmal or persistent AF (Figure 1A), which was comparable to the success rates reported previous. However, the 5-year success rate in patients with persistent AF was only 36.4%, which was significantly lower than that in patients with paroxysmal AF (63.2%; Figure 2). Chan et al. (7) reported the 5-year success rate in patients with non-paroxysmal AF was 30% and 45% by using first-generation and second-generation CB, respectively. Studies reported that the 5-year success rate in patients with paroxysmal and persistent AF was 59.6% and 46.9% by using second-generation CB (15). In addition to PV triggers, other mechanisms, such as rotors and non-PV foci, also play an important role in leading to AF (11, 16, 17). Therefore, besides achieving PVI, additional ablative strategies (additional superior vena cava, posterior wall or left atrial roof line isolation) may be required for patients with persistent AF, which could reduce the AF recurrence (17–19).

Interestingly, we found that the rate of AF recurrence mainly occurred within the first year after the index procedure, and then it stabilized (Figure 1B). A previous study showed similar phenomenon that the rate of decline in freedom from AF recurrence stabilized after the initial 12 months (about 30% during the first year and about 17% during the following 4 years) (12). Similarly, Vogt et al. (20) reported that the rate of decline in freedom from AF recurrence was steepest within the initial 12 months post ablation with first-generation CB (22.4%), which was about twice than that after the initial 12 months (10.6%). In present study, 15 out of 18 patients (88.3%) who underwent repeat ablation had PV reconnection, and these patients experienced AF recurrence after a median time of 7.5 [3.0, 21.8] months from the initial procedure. Moreover, it was recently indicated that the mean time to AF recurrence after initial PVI was 6 ± 6 months for patients with PV reconnection during repeat ablation (16). A meta-analysis showed that 85.5% had at least 1 PV reconnected among patients with AF recurrence (21). We therefore speculated that PV reconnection might be an important factor for AF recurrence within the first year. Some studies reported that the rate of AF recurrence was 3–19% in patients undergoing repeat ablation of reconnected PVs (22–24). The PRESSURE randomized controlled trial revealed that routine repeat ablation in patients with PV reconnection provided significant improvements in freedom from AF recurrence, AF burden, and quality of life (25). Therefore, patients could potentially benefit from early repeat ablation, especially for those with PV reconnection.

### Predictors of Five-Year Recurrence After Cryoballoon Ablation of Atrial Fibrillation

AF recurrence after CB ablation procedure is still focus of attention, and it is important to determine the predictors that affect AF recurrence. Although many predictors of AF recurrence in patients undergoing CB ablation for treatment of AF have been described in previous studies, independent predictors of 5-year AF recurrence remain limited (6, 20, 26, 27). In studies of 5-year follow-up post ablation of AF with CB, different indictors based on LAD were reported to be the independent predictor for AF recurrence (12, 13). However, LAD was not found to be associated with AF recurrence in our study, which could be attributed to the exclusion of patients with LAD ≥ 50 and subsequent smaller mean LAD among included participants. In our study, only persistent AF (HR 1.72, 95%CI 1.028–2.854, *P* < 0.05) identified to be independent predictors for 5-year AF recurrence after a single CB ablation procedure (Table 3). After a median follow-up time of 34 months post ablation of AF with first-generation CB, Bohó et al. (27) also found only persistent AF (HR 1.97, 95% CI: 1.24–3.13, *P* = 0.006) was the independent predictor for AF recurrence. A meta-analysis demonstrated that persistent AF (HR 2.44, 95% CI: 1.30–4.58, *P* < 0.006) was an independent predictor of late recurrence after CB ablation (26). As we discussed above, only achieving PVI by CB might be less sufficient for patients with persistent AF.

### Safety

Previously published studies showed that PNI was the most frequent procedural complication associated with CB ablation, occurring over a range of 2.7–12.7% (3, 28, 29). In our study, PNI was also the most common complication, with an incidence of 3.5%. In addition, all patients developed PNI were performed by using 23-mm CB and recovered within one year post the procedure. Actually, the occurrence of PNI has several certain characteristics. Firstly, compared with 28-mm CB, ablation with 23-mm CB produces a higher incidence of PNI (20, 27). Secondly, the PNI is reversible to some extent, which can generally recover within the procedure or several days post procedure, or rarely within one year in some cases (3). Thirdly, the incidence of PNI will decrease with the enrichment and accumulation of experience (10). To avoid PNI, our experience was to avoid placing the CB too deep into PVs, and to pace the phrenic nerve continuously and steadily when starting to freeze the right-side PVs.

Although five patients developed mild pericardial effusion, there was no fatal complications like stroke, atrio-esophageal fistula or death occurred. The incidence of peripheral vascular puncture complications was 2.3%, which were also comparable to that reported previously (20, 27, 28). No late or unexpected complications were detected during the 5-year follow-up.

### Limitation

There are several potential limitations to this study. Firstly, this study is a single-center investigation, and the population size is relatively small. Secondly, all included patients were performed by using the first-generation CB, hence, some results could not be applied to those by using the second-generation CB. Thirdly, our follow-up was based on intermittent rhythm monitoring *via* 24-h Holter monitor or serial electrocardiogram recordings rather than intensive monitoring *via* continuous transtelephonic monitoring or implantable loop recorder, therefore, some non-sustained or asymptomatic episodes of AF could not be recognized which could underestimated the recurrence rate (30). Fourthly, although there was no statistical difference between AF recurrence and LAD, the patients with larger LAD tended to have higher AF recurrence rate. Patients with LAD ≥ 50 mm were excluded, which might introduce selection bias and result in inability of observing the significant difference between LAD and AF recurrence. Last but not least, the number of patients with persistent AF is too small, which may occasionally result in a higher recurrence rate among those patients, and further cause a certain bias when analyzing predictors of AF recurrence.

## Conclusion

In conclusion, PVI using CB ablation was safe and effective with an acceptable complication rate of 7.8% and a 5-year success rate of 59.4% in a Chinese population with AF, and persistent AF was shown to be an independent predictor for 5-year AF recurrence after a single CB ablation procedure.

## Data Availability Statement

The raw data supporting the conclusions of this article will be made available by the authors, without undue reservation.

## Ethics Statement

The studies involving human participants were reviewed and approved by the Ethics Committee of the Fuwai Hospital. The patients/participants provided their written informed consent to participate in this study.

## Author Contributions

XC, YX, and JL were responsible for the conception and design of the study. XC, YX, YL, XL, PF, and JL contributed substantially to the data acquisition. XC, YX, YL, XL, CW, and YC were part of the data analysis committee. XC, YX, YL, XL, CW, YC, PF, and JL contributed to the data interpretation. YX, YL, and XL were responsible for the acquisition of survival data and comorbidities. XC and YX drafted the manuscript. JL was responsible for the acquisition of funding. All authors contributed substantially to the critical revising of the manuscript for important intellectual content, final approval of the version to be published, and agreed to be accountable for all aspects of the work.

## Conflict of Interest

The authors declare that the research was conducted in the absence of any commercial or financial relationships that could be construed as a potential conflict of interest.

## Publisher’s Note

All claims expressed in this article are solely those of the authors and do not necessarily represent those of their affiliated organizations, or those of the publisher, the editors and the reviewers. Any product that may be evaluated in this article, or claim that may be made by its manufacturer, is not guaranteed or endorsed by the publisher.
